# Microbial biomarkers for immune checkpoint blockade therapy against cancer

**DOI:** 10.1007/s00535-018-1492-9

**Published:** 2018-07-12

**Authors:** Keishi Adachi, Koji Tamada

**Affiliations:** Department of Immunology, Yamaguchi University Graduate School of Medicine, 1-1-1 Minami-Kogushi, Ube, Yamaguchi 755-8505 Japan

**Keywords:** Immunotherapy, PD-1, Cancer, Biomarker, Enteric bacteria

## Abstract

Three major standard treatments, i.e., surgery, chemotherapy, and radiotherapy, were traditionally applied to the treatment of cancer and saved many patients. Meanwhile, clinical studies as well as basic research of immunotherapy are being actively conducted for intractable or advanced malignancies that cannot be cured by the conventional standard treatments. Remarkable therapeutic efficacies have been recently reported in clinical trials on some cancer types, and immunotherapy is now being recognized as the “fourth” standard therapy against cancer. In particular, immune checkpoint inhibitor therapy (ICI) has demonstrated the effectiveness of immunotherapy through large-scale randomized clinical trials, leading to the paradigm-shift in cancer treatment. Immune checkpoint molecules transduce co-inhibitory signals to immunocompetent cells including T cells, and crucially contribute to the formation of an immunosuppressive microenvironment in tumor tissues, which intrinsically confers the treatment resistance. Programmed death-1 (PD-1, CD279) is one of the typical immune checkpoint molecules. Anti-tumor therapies targeting PD-1 and its ligands had been developed and approved in many countries, and various studies utilizing clinical specimens are currently progressing. In this review, we provide an overview of the biomarkers based on the analysis of enteric microbiota that correlate with the clinical efficacy/inefficacy of PD-1-based therapy.

## Introduction

**Fig. 1:**
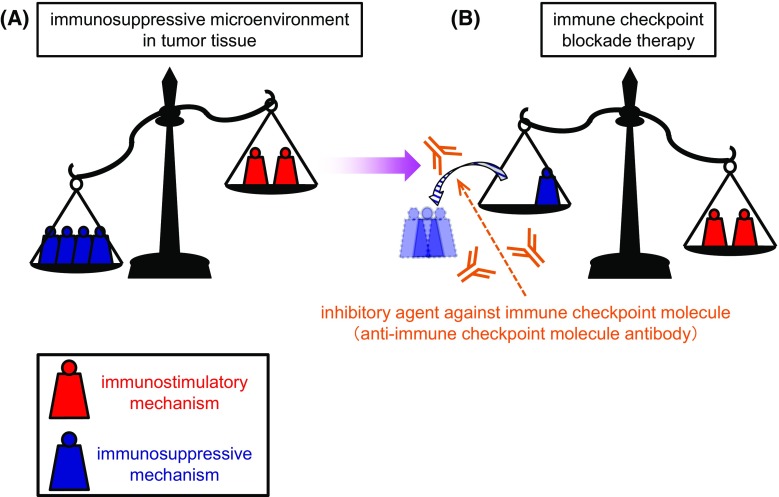
Conceptual diagram of immune checkpoint blockade therapy. **a** In tumor microenvironment, the immunological balance is conspicuously biased toward inhibitory-dominant side. The aim of cancer immune therapies is to make the balance of the host immunity biased toward stimulatory-dominant side. **b** In immune checkpoint blockade therapy, the balance is shifted by “decreasing or removing the weights from the inhibitory side” with inhibitory agent against immune checkpoint molecule such as blocking antibodies

Immune checkpoint molecules regulate the host immune system by transducing immunosuppressive co-signals to immunocompetent cells [1–5]. The most representative ones are CTLA-4 (cytotoxic T-lymphocyte-associated protein 4, CD152) and PD-1/PD-L1 (CD274), PD-L2 (CD273) [6–13]. By being expressed “at appropriate timing” and on “appropriate cell types”, they play a major role in preventing overactivation of host immune system and in keeping immunological homeostasis and tolerance [1, 2, 5]. Meanwhile, it has been reported that immune checkpoint molecules are aberrantly expressed in tumor tissues [3, 14–16]. As a result, a potent immunosuppressive milieu is generated in tumor tissues, which is one of the major causes of the treatment resistance in many cancer types. The aim of ICI is to disarm or mitigate the immunosuppressive mechanisms in the tumor microenvironment with inhibitory agents targeting immune checkpoint molecules (Fig. 1) [2, 5, 17]. To date, not only anti-PD-1 antibodies (e.g., nivolumab and pembrolizumab) but also anti-PD-L1 (e.g., atezolizumab, avelumab, and durvalumab) and anti-CTLA-4 (e.g., ipilimumab) antibodies have been approved worldwide as therapeutic medicines for multiple cancer types [18–23].

## Biomarkers associated with clinical efficacy of ICI

Regardless of its clinical success, ICI is facing several problems to be overcome. One of the most crucial issues is identification of biomarkers which correlate with the clinical benefits or with the adverse events [3, 24–26]. For example, the response rates of ICI using a single agent against melanoma are about 20–40% [24, 27–30]. Therefore, establishment of biomarkers enabling appropriate selection of patients is a critical issue from the viewpoint of healthcare cost matter as well as of patients’ quality of life. To date, several biomarkers for predicting the effectiveness or ineffectiveness of ICI have been reported, and many of them rely on cellular or molecular examinations such as immunohistochemistry and flow cytometry. In anti-PD-1 or anti-PD-L1 antibody therapy against several cancer types, some pathological features as follows are correlated with the response rates [3, 18, 20, 31–38]:

- Infiltration of T cells into or around the tumor tissue.
- Expression of PD-L1 inside the tumor tissue.
- Expression of PD-L1 on immune cells infiltrating to the cancer tissues.

Unfortunately, it is claimed that those biomarkers are not universally applicable to many cancer types. Furthermore, many exceptional cases, such as ineffectiveness on PD-L1-positive tumors and effectiveness on PD-L1-negative tumors, are reported, and hence, they cannot perfectly predict the effectiveness or ineffectiveness [24, 39–43]. Therefore, the development of the biomarkers with a new approach has been pursued.

## Bacterial biomarkers for PD-1-based therapy against melanoma

The evolution and popularization of next-generation sequencer led to technological innovations in the studies of enteric microbiota, and uncovered that enteric microbiota profoundly affects host immune system [44–48]. This prompted to conduct investigations focusing on the relationship between efficacy of ICI and intestinal flora, and some intriguing findings have been reported mainly in melanoma. The finding that intestinal flora affected the efficacy of a therapy targeting PD-1/PD-L1 axis was initially reported with an experimental murine melanoma model [49]. It was demonstrated that oral administration of *Bifidobacterium* species including *B. breve* and *B. longum* resulted in an improved tumor control without additional treatments, and that the improvement was further augmented in combination with anti-PD-L1 antibody treatment. Tumor-specific T cells increased in the tumor tissue as well as the periphery in those mice, and the depletion of CD8^+^ T cells canceled the therapeutic effects. Furthermore, the *Bifidobacterium* feedings enhanced the capacity of dendritic cells to stimulate CD8^+^ T cells. These suggested that the colonization of *Bifidobacterium* species modulated dendritic cell activation, leading to the exertion of anti-tumor effects via evoking T cell immunity [49]. Previous studies demonstrated that some species of *Bifidobacterium* had a potential to modulate DC activation directly and to influence T-cell responses [50–55]. Although innate immune systems including Toll-like receptors should be involved [56–61], heat inactivation of those bacteria wiped out the anti-tumor effects after their oral administration [49]. This suggested that live bacteria were indispensable and that bacterial components alone were insufficient.

Based on the results described above, analyses using feces of metastatic melanoma patients who had received anti-PD-1 antibody therapies were conducted by the same group at University of Chicago [62]. In that cohort study, stool specimens were collected from 42 metastatic melanoma patients prior to the anti-PD-1 antibody treatment, and the correlations between the compositions of intestinal flora and the therapeutic efficacies were examined. It was demonstrated that eight bacterial species, including *Bifidobacterium longum*, *Collinsella aerofaciens*, and *Enterococcus faecium*, were enriched in the responders to the PD-1-based therapy as compared to the non-responders. On the other hand, two bacterial species, *Ruminococcus obeum* and *Roseburia intestinalis*, were found to be more abundant in the non-responders than the responders [62]. Fecal microbiome transplantation into germ-free mice revealed that feces of the responders, but not that of the non-responders, had a capacity to control tumor growth. Fecal transplantation from the responder increased tumor-specific T cells not only in the spleen but also in the tumor tissue of the mice as compared to that from the non-responder, indicating that colonization of the beneficial bacteria primed tumor antigen-specific immunity locally as well as systemically [62]. Furthermore, the experimental fecal transplantation from the responders into mice augmented the therapeutic effects of anti-PD-L1 antibody, whereas that from the non-responders abrogated the effects. Collectively, colonization of several bacterial species including *Bifidobacterium longum*, which was found also in the murine study described above, was associated with anti-tumor efficacies of PD-1-based therapy in the cohort of this study [62].

In another cohort study conducted at The University of Texas MD Anderson Cancer Center, fecal samples were collected from 112 metastatic melanoma patients before and after the anti-PD-1 antibody treatment, and the correlations between the diversity and compositions of the intestinal flora and the clinical responses were analyzed [63]. The diversity of microbiota of the responders of the therapy was profoundly higher than that of the non-responders, resulting that the patients with high diversity had significantly longer progression-free survival (PFS) than those with intermediate or low diversity [63]. It was also uncovered that Ruminococcaceae family and *Faecalibacterium* genus were enriched in fecal microbiota of the responders, whereas Bacteroidales was abundant in those of the non-responders. Consistent with this finding, patients with high *Faecalibacterium* abundance displayed longer PFS than those with lower abundance, while patients with high Bacteroidales abundance had shorter PFS than those with lower abundance [63]. Immunohistochemical analyses of the tumor tissues revealed that the infiltration of CD8^+^ T cells into the tumor and the abundance of the *Faecalibacterium*, the Ruminococcaceae, and the Clostridiales in the gut were positively correlated [63]. Moreover, in the systemic circulation, the patients with the high abundance of the *Faecalibacterium*, the Ruminococcaceae, and the Clostridiales displayed high frequencies of CD8^+^ T cells and effector CD4^+^ T cells. In contrast, the patients with the high abundance of the Bacteroidales exhibited high frequencies of immune-suppressive cell populations such as regulatory T cells (T_reg_) and myeloid-derived suppressor cells in the systemic circulation [63]. Fecal microbiome transplantation into germ-free mice revealed that feces of the responders significantly suppressed tumor growth as compared to that of the non-responders. Moreover, the experimental fecal transplantation from the responders into mice improved the therapeutic efficacy of anti-PD-L1 antibody treatment, although that from the non-responders worsened [63]. Tumor tissues of the mice receiving the feces from the responders exhibited higher levels of CD8^+^ T cell infiltration and of PD-L1 expression than those form the non-responders, suggesting that colonization of the beneficial bacteria in the gut would generate the immunologically “hot” microenvironment in the tumor tissues. Moreover, high frequency of innate immune effector cells expressing CD45, CD11b, and Ly6G [64], and low frequency of myeloid suppressor cells expressing CD11b and CD11c [65] were observed in the tumor tissues of mice receiving the feces of the responders as compared to those of non-responders. In contrast, the mice receiving the feces of the non-responders displayed higher frequencies of T_reg_ in their spleens than those of the responders [63]. Consistent with the aforementioned results in another cohort, these results indicated that colonization of specific bacteria in the gut would influence anti-tumor immunity not only systemically but also locally. Altogether, in PD-1-based therapy on the cohort of this study, enrichment of Ruminococcaceae family and *Faecalibacterium* genus was correlated with the effectiveness, whereas that of Bacteroidales was correlated with the ineffectiveness [63].

## Bacterial biomarkers for PD-1-based therapy against cancers other than melanoma

In the cohort study conducted at three clinical sites in France, the correlation between antibiotic treatment and the efficacy of PD-1-based therapy was investigated on 249 patients with epithelial cancers including non-small cell lung cancer (NSCLC), renal cell carcinoma (RCC) and urothelial carcinoma [66]. The patients who were treated with antibiotics before or after the antibody therapy displayed shortened PFS and overall survival as compared with those who were not treated with antibiotics, and this was also the case in experimental murine models. These suggested that dysbiosis might affect the efficacies of anti-PD-1/PD-L1 antibody therapy [66]. Based on those results, fecal microbiome analyses were conducted on the NSCLC and RCC patients. By comparing between the responders and non-responders of the PD-1-based therapy, the intestinal bacterium most significantly associated with beneficial therapeutic responses in both NSCLC and RCC patients was *Akkermansia muciniphila* [66]. Interestingly, the duration of PFS was positively correlated with the IFN-γ production from peripheral blood CD4^+^ T cells and CD8^+^ T cells in response to *A. muciniphila* but not to TCR ligation. This might indicate that T cell responses specific to *A. muciniphila*, but not non-specific bystander responses, had some relationships with anti-tumor effects. Fecal microbiome transplantation from the responders of the PD-1-based therapy into antibiotic-treated or germ-free mice restored the anti-tumor efficacy of anti-PD-1 antibody treatment although that from the non-responders did not [66]. Upon the fecal transplantation from the responders, the accumulation of CXCR3^+^ CD4^+^ T cells, which is a characteristic feature of Th1 [67], in the tumor tissues as well as the up-regulation of PD-L1 on CD4^+^ T cells in the spleens were observed. Mono-colonization with *A. muciniphila* on the tumor-bearing mice treated with antibiotics restored the sensitivity to anti-PD-1 antibody treatment. Furthermore, oral administration of *A. muciniphila* into the mice that received the fecal transplantation from the non-responders ameliorated the efficacy of the anti-PD-1-based therapy. Immunohistochemical examination exhibited that in the tumor tissues of mice co-treated with *A. muciniphila* and anti-PD-1 antibody, but not in those treated with the antibody alone, the ratio of CD4 to FoxP3, a definitive transcription factor for T_reg_ [68], was increased, suggesting that the immune-stimulatory condition was induced in the tumor after the combinatory treatment. Furthermore, *A. muciniphila* stimulated dendritic cells in vitro to produce IL-12, which is the crucial cytokine for the differentiation to Th1 [69]. The neutralization of IL-12 or IFN-γ with the specific antibodies eliminated the in vivo anti-tumor efficacy by the co-treatment of anti-PD-1 antibody and *A. muciniphila*. Taken together, colonization of *A. muciniphila* would play an important role in the efficacy of the therapy targeting PD-1/PD-L1 axis against some types of cancers through the induction of Th1 responses [66].

## Future perspectives of biomarkers

At present, a large number of clinical trials of ICI are ongoing worldwide, and analyses using clinical specimens including feces are also actively being conducted. In this review, we focused on enteric microbiota that could be biomarkers correlating with the efficacy/inefficacy of PD-1-based therapies. Interestingly, it has also been reported that the efficacy of CTLA-4 blockade therapy against melanoma is associated with some enteric bacteria, *Bacteroides fragilis* and/or *B. thetaiotaomicron* [70]. Nonetheless, it is unlikely that those bacteria alone can be the complete biomarker universally applicable to many cancer types. Besides the intestinal bacteria described here, several biomarkers predicting clinical benefits or adverse events based on genetic analysis have been reported so far, listed as follows:

- Tumor cells

1. Mutation burden leading to the generation of neoantigens [71, 72].
2. Activation of Wnt/β catenin signals [73].
3. Loss-of-function mutation of the genes related to DNA mismatch repair system [74, 75].
4. 3′-UTR disruption of PD-L1 gene leading to the aberrant expression of PD-L1 [76].

- Other than tumor cells

1. TCR repertoire of peripheral T cells [77, 78].
2. TCR repertoire of tumor-infiltrating T cells [79, 80].

**Fig. 2:**
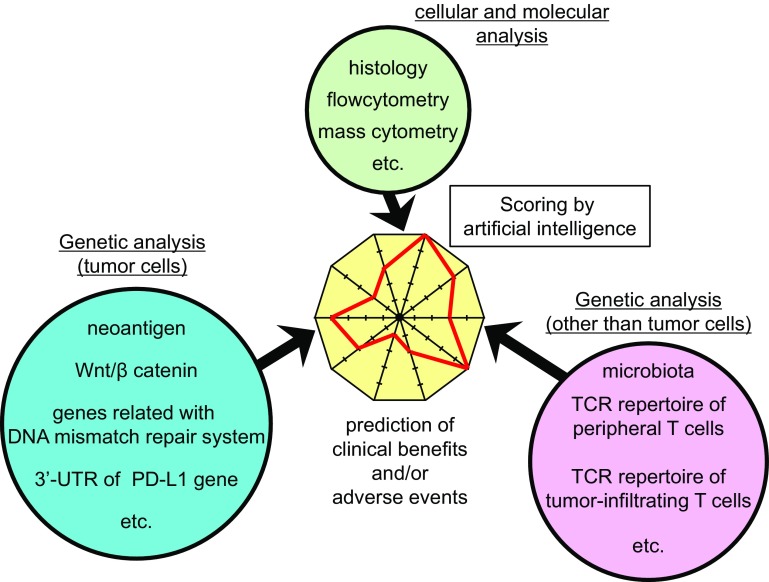
Future perspectives of biomarker for immune checkpoint blockade therapy. It is expected that more accurate prediction of clinical benefits and/or adverse events will be realized by comprehensive integration of multiparametric biomarkers for individual patients, containing histological and cytometric examination and genetic analysis of tumor cell and non-tumor cells. In that process, artificial intelligence would play a crucial role in the scoring procedure for the selection of optimal patients or therapy

Henceforth, it is anticipated that more accurate prediction of clinical benefits and/or adverse events will be realized by comprehensive integration of multiparametric biomarkers for individual patients, and that artificial intelligence would play a crucial role in the selection of optimal patients or therapy (Fig. 2).

At present, the precise molecular mechanisms by which some specific bacteria described above affect the anti-tumor efficacy remain unsolved [81, 82]. For example,

- the speciality of those bacteria: do they have special component(s) and/or secreted material(s) that do not exist in other bacteria?
- the specificity to tumor: do they have T cell epitope(s) resembling tumor-associated antigen(s) and/or neoantigen(s), and induce the cross-reaction to tumor?
- the difference in bacterial species among the cohorts: does the difference in the basal intestinal microbiota due to ethnic groups, dietary habits, living environments, and so on, affect the difference of those beneficial bacteria?
- the “remote control” of anti-tumor immunity: how does the colonization of those bacteria in intestinal tract modulate the efficacies of ICI at distal tumor sites?

By elucidating the issues, some enteric bacteria or their derivatives, i.e., probiotics, can be candidate agents for the novel combination therapy utilizing immune checkpoint inhibitors [82, 83].
